# Ocean Acidification Affects Prey Detection by a Predatory Reef Fish

**DOI:** 10.1371/journal.pone.0022736

**Published:** 2011-07-28

**Authors:** Ingrid L. Cripps, Philip L. Munday, Mark I. McCormick

**Affiliations:** 1 School of Marine and Tropical Biology, James Cook University, Townsville, Australia; 2 Australian Research Council Centre of Excellence for Coral Reef Studies, James Cook University, Townsville, Australia; Hungarian Academy of Sciences, Hungary

## Abstract

Changes in olfactory-mediated behaviour caused by elevated CO_2_ levels in the ocean could affect recruitment to reef fish populations because larval fish become more vulnerable to predation. However, it is currently unclear how elevated CO_2_ will impact the other key part of the predator-prey interaction – the predators. We investigated the effects of elevated CO_2_ and reduced pH on olfactory preferences, activity levels and feeding behaviour of a common coral reef meso-predator, the brown dottyback (*Pseudochromis fuscus*). Predators were exposed to either current-day CO_2_ levels or one of two elevated CO_2_ levels (∼600 µatm or ∼950 µatm) that may occur by 2100 according to climate change predictions. Exposure to elevated CO_2_ and reduced pH caused a shift from preference to avoidance of the smell of injured prey, with CO_2_ treated predators spending approximately 20% less time in a water stream containing prey odour compared with controls. Furthermore, activity levels of fish was higher in the high CO_2_ treatment and feeding activity was lower for fish in the mid CO_2_ treatment; indicating that future conditions may potentially reduce the ability of the fish to respond rapidly to fluctuations in food availability. Elevated activity levels of predators in the high CO_2_ treatment, however, may compensate for reduced olfactory ability, as greater movement facilitated visual detection of food. Our findings show that, at least for the species tested to date, both parties in the predator-prey relationship may be affected by ocean acidification. Although impairment of olfactory-mediated behaviour of predators might reduce the risk of predation for larval fishes, the magnitude of the observed effects of elevated CO_2_ acidification appear to be more dramatic for prey compared to predators. Thus, it is unlikely that the altered behaviour of predators is sufficient to fully compensate for the effects of ocean acidification on prey mortality.

## Introduction

Growing evidence suggests that ocean acidification, caused by rapidly increasing anthropogenic CO_2_ emissions, will have significant and widespread impacts on marine life [1]–[3]. Based on current emission trajectories, atmospheric CO_2_ concentrations are predicted to reach 730–1,020 parts per million (ppm) by the end of the century [4]. Corresponding increases in CO_2_ dissolved in the ocean may cause a reduction of 0.3–0.4 units in oceanic pH compared to current-day levels [5]–[6]. This departure from current-day pH levels would occur at a faster rate than has been seen at any time over the past two million years, potentially limiting the abilities of populations to adapt to such a rapid change [6]–[7].

The potential impact of ocean acidification on the growth and survival of marine calcifiers is well established [8]–[13], however the likely effects of elevated CO_2_ and reduced pH on non-calcifying organisms, such as marine fishes, is still poorly understood. Recent research has demonstrated that the ability of larval fish to detect ecologically important cues is hindered by ocean acidification [14]–[17]. Larval clownfish exposed to elevated CO_2_ levels were unable to distinguish olfactory cues for suitable adult habitat, parental scent [14] and predator odour [15]. This impairment of predator cue recognition, combined with changes in behavioural boldness, was associated with a 5–9 times higher rate of mortality from predation for newly settled larvae that were reared in elevated CO_2_ compared with controls [18]. Such dramatic changes in mortality could have potentially serious implications for population replenishment and ecosystem diversity [18]. However, the effects of ocean acidification on predators, which are the determinant of prey mortality rates, are yet to be addressed. If ocean acidification affects both predators and prey equally, there may be no net effect on mortality rates.

Predators are vital for the maintenance of ecosystem health and for structuring marine communities [19]–[21]. Predation alters the community dynamics of marine populations through the reduction of prey abundance [22]–[23] and may influence species diversity [24]–[25]. However, not all predators are functionally equal, with different species exerting effects on particular species or size classes of prey. On coral reefs, predation has a highly significant effect on the recruitment rates of newly settled fishes. Upon settling to the benthos, larvae are subjected to high rates of mortality, with up to 60% of recruits consumed within the first two days of settlement [20], [26]. Small reef-associated predatory fishes (meso-predators) that feed opportunistically on recruits are the major agents of this mortality [27]–[29].

Various sensory systems are employed in the feeding process of meso-predators, which involves; searching, detection, capture and ingestion [30]. Predators are particularly reliant on chemical and visual cues to detect prey [31]. Visual cues can be limited in aquatic environments due to habitat complexity, turbidity from suspended sediments and low light conditions [32]. Additionally, the solvent properties of water and the high persistence of chemical cues render the sense of olfaction particularly useful in marine environments [33]–[35]. Olfactory cues are likely to be important for meso-predators due to the small spatial scales over which they act and the topographic complexity of coral reefs. Particularly important for both predators and prey are chemical alarm cues (skin extracts), released upon mechanical damage to the skin of prey species [36]. Prey seek shelter and reduce activity levels when alarm cues are detected, whereas predators are directly attracted to chemical alarm cues of their prey [37]–[38].

Predation events involve two parties, predator and prey. While evaluations have been conducted on the response of prey species to ocean acidification [14]–[16], [18], research is yet to address the threat of ocean acidification from a predator's perspective. Therefore, in this study we aimed to investigate the influences of ocean acidification on the behaviour of a common coral reef meso-predator; the brown dottyback *Pseudochromis fuscus* (Pseudochromidae). Firstly, the effects of elevated CO_2_ on the olfactory abilities of *P. fuscus* was evaluated using olfactory pairwise choice tests. The response of predators to prey skin extract was assessed following exposure to control (current-day CO_2_) or one of two elevated CO_2_ levels (∼600 µatm or ∼950 µatm CO_2_) which match low- and high-end predictions of conditions that could occur in the ocean by 2100 according to future climate change scenarios [4], [39]. Secondly, the effects of similar levels of elevated CO_2_ on the activity levels and feeding behaviour of *P. fuscus* was investigated using behavioural assays. Understanding the effects on both predator and prey fish is crucial for determining the potential impacts of future ocean acidification on this relationship.

## Materials and Methods

### Study site and specimen collection

This study was conducted at Lizard Island Research Station (LIRS; 14°40′S, 145°28′E) in the northern section of the Great Barrier Reef (GBR) between March and April 2010. Experiments used the flow-through seawater system at LIRS, and the study species was collected from the nearby fringing reefs. The model predator species was *P. fuscus*, which is a common meso-predator on the GBR. *P. fuscus* occurs in high densities at Lizard Island, and is known to have a significant effect on population dynamics of common damselfish species by opportunistically feeding on recently settled juveniles [29]. *P. fuscus* were collected from shallow water reefs (∼6 m) in the Lizard Island lagoon with the aid of a mild anesthetic mixture of ethanol, clove oil and seawater [40] and caught using hand-nets. Captured fish were placed in large plastic bags and transported to the research station. Fish were transferred to replicate 35 L aquariums supplied with a continuous flow of seawater diffused with one of three different CO_2_ treatments (see ocean acidification system). Prior to experimentation, *P. fuscus* (Total Length (TL); 64.07±1.31 mm; mean ± SE) were kept in treatment for four-seven days. Preliminary trials indicated that treatment with enriched CO_2_ for this period of time was sufficient to alter olfactory-mediated behaviour, as observed in studies conducted on different species [18]. Two fish were held in each aquarium, divided by a plastic mesh barrier in the centre of the tank and all were fed INVE Aquaculture Nutrition pellets once daily.

Juvenile lemon damselfish, *Pomacentrus moluccensis*, (Pomacentridae) were chosen as the ‘prey’ species from which damaged-skin-extracts were sourced as they are known prey of *Pseudochromis fuscus* [29], [41] and have been used in previous chemical alarm cue experiments. *Pomacentrus moluccensis* were collected by using the same methods described above, held in 57 L tanks and fed *ad libitum* with freshly hatched *Artemia* nauplii. All procedures were approved by James Cook University Animal Ethics Committee (A1511).

### Seawater system

*P. fuscus* were held for four – seven days in aquariums containing either control seawater or water enriched with CO_2_. Seawater was pumped from the ocean into 3×60 L sumps where it was diffused with air (control) or CO_2_ to achieve a pH of approximately 8.15 (control), 8.00 (mid) or 7.90 (high). A pH-controller (Tunze Aquarientechnik, Germany) was attached to each of the CO_2_ treated sumps to maintain pH at the desired level (8.00 or 7.90). A solenoid injected a slow stream of CO_2_ into a powerhead at the bottom of the sump whenever the pH of the seawater rose above the set point. The powerhead rapidly dissolved CO_2_ into the seawater and also served as a vigorous stirrer. Using this method it was possible to constantly maintain pH within ±0.05 units of the desired value. Equilibrated seawater from each sump was supplied at a rate of ∼500 ml sec^−1^ to four replicate 35 L aquariums, each housing two *P. fuscus*, as described above. To maintain oxygen levels and the required *p*CO_2_ levels, aquariums were individually aerated with air (control ∼400 µatm) or CO_2_-enriched air (∼600 µatm or ∼950 µatm). The concentration of CO_2_-enriched air was controlled by a scientific-grade pressure regulator and precision needle valve and measured continuously with an infrared CO_2_ probe (Vaisala GM222).

Temperature and pH_NBS_ of each aquarium was checked twice daily using a HQ40d pH meter (Hach, Colorado, USA), calibrated bi-weekly with fresh buffers (Merck). Total alkalinity of seawater (TA; µmol.kg^−1^SW) was estimated by Gran titrations from a total of 39 water samples. Average seawater *p*CO_2_ was calculated with these parameters in the program CO2SYS using the constants of Mehrbach et al (1973) refit by Dickson and Millero (1987). *p*CO_2_ estimated by seawater chemistry during olfactory tests were 450.63±0.64 µatm (control; mean ± SE), 630.09±0.78 µatm (mid) and 948.94±0.78 µatm (high; Table 1). *p*CO_2_ estimates during the activity level and feeding behaviour tests were 444.02±0.44 µatm (control), 607.34±1.28 µatm (mid) and 925.49±0.69 µatm (high; Table 1).

**Table 1:** Seawater parameters for the olfactory and behavioural experiments. Values are means (± SE).

Experiment	Treatment	pH_NBS_	Temp.(°C)	Salinity (ppt)	Total Alkalinity (µmol.kg^−1^SW)	*p*CO_2_ (µatm)
Olfaction	Control	8.16 (0.01)	27.5 (0.01)	34.8	2264.65 (2.58)	450.63 (0.64)
	Mid	8.03 (0.01)	27.4 (0.1)	34.8	2264.65 (2.58)	630.09 (0.78)
	High	7.88 (0.01)	27.5 (0.1)	34.8	2264.65 (2.58)	948.94 (0.74)
Activity	Control	8.14 (0.01)	28.0 (0.03)	34.8	2264.65 (2.58)	444.02 (0.44)
	Mid	8.05 (0.01)	28.2 (0.05)	34.8	2264.65 (2.58)	607.34 (1.28)
	High	7.87 (0.01)	27.5 (0.1)	34.8	2264.65 (2.58)	925.49 (0.69)

### Experimental protocol

#### Experiment 1 – Response of Pseudochromis fuscus to prey skin extracts

Pairwise olfactory choice trials were run in a two channel chamber (600×250×110 mm), with a water depth of 90 mm. A centered 450 mm plastic barrier divided the chamber into two compartments of equal size. The small area along the back wall of the chamber was used as an acclimation area and separated from the rest of the chamber using 5 mm rigid mesh to maintain water flow through the chamber. Seawater was pumped directly from the ocean to a common reservoir, where it was gravity fed into two identical water outlets at the rear of the chamber at 7.21±0.77 L.min^−1^, and exited along outlets at the front of the chamber. Rigid mesh (5 mm) was placed directly in front of the outlets to aid in laminar flow and prevent concealment of *P. fuscus*. Two identical shelters were placed in both compartments, allowing for the water to move through the hide and minimizing disruption to the flow. A 1.5 m long plastic 4 mm tube was attached to each water outlet, just below the surface of the water. Cues were injected via this tube allowing for their dispersion through the compartment. One channel of the chamber received the prey damaged-skin-extracts from juvenile *Pomacentrus moluccensis*. The other compartment received an equal quantity of seawater control with no additional chemical cues (i.e. a control). Dye trials indicated stimulus flow was even in the chamber compartments, flowing through the chambers and exiting at the front. The stimulus moved through the compartment at 23±2.5 mm.sec^−1^ and the chamber had been flushed after 4.5 min. All trials were undertaken at a constant temperature of 27°C. Fish were not fed for two days prior to trials to standardize for satiation. Control trials, where untreated seawater only was injected into both channels were randomly interspersed among trials with skin extracts. These trials confirmed that the fish used each compartment in approximately equal frequency when no additional cue was present.

At to the start of each trial, a fish was placed in the barricaded area at the front of the chamber and allowed to acclimate for a minimum of four hours. Before the removal of the barrier, 60 ml of seawater was drawn through each of the tubes and discarded to remove stagnant water. Another 60 ml of seawater was drawn through the injection tubes and retained. 15 ml of the stimulus was then injected into each tube simultaneously, followed by the 60 ml of seawater previously removed, to flush the stimuli into the respective chamber compartments. The barrier was slowly removed and the behavioral observations commenced. Trials occurred over eight min and stimuli were re-injected at four min intervals. After the trial ceased, fish were once again retained in the acclimation area, and chambers were left to flush for approximately one hour. The compartments into which the stimuli were injected were swapped and the trial was repeated. Following the injection of the stimuli and the removal of the barrier, the location of the fish within the chamber was recorded every five seconds. Only the time spent in the channels of the chamber were included in the analysis as fish from all treatment groups within the control trials (SW vs SW) and treatment (prey skin extract vs SW) trials spent on average, less than 0.25 of their time in the acclimation area. As this time was minimal in the total time of the trial, removal of this period from the analysis was deemed to be acceptable. This exclusion was also due to fact that mixing of the cues could not be ruled out, as the water flowed out of the chamber. Dye trials indicated that some mixing could have been occurring in this small rear section of the chamber as the water moves out of the draining holes. Therefore, the time the fish were spending in this area was not necessarily indicative of a choice of cue, as fish could have been exposed to both the untreated seawater and the prey skin extract.

The sequence of the olfactory preference tests for the control and CO_2_ treated fish were randomised throughout the duration of the experiment. Previous investigations, on different species, have indicated that testing in control water did not yield any differences to testing in CO_2_ treated water [15]–[16] and thus all olfactory trials were conducted using control water. Observations were undertaken from behind a black barrier to minimise disturbance to fish.

#### Preparation of experimental stimuli

Prey cues were prepared using skin extracts from juvenile *Pomacentrus moluccensis*. Fish were euthanised by a quick blow to the head and placed in a clean petri dish. Vertical incisions were made along the flank of each fish using a scapel blade, and the specimen was rinsed in 15 ml of seawater. Two damselfish (TL; 22.04±0.65 mm; mean ± SE) were used for each skin extract solution, with eight superficial cuts of similar size, made along each side of the flank to standardize for size.

#### Experiment 2 – Activity levels and feeding behaviour of *Pseudochromis fuscus*

Baseline activity levels of *P. fuscus* were recorded to discern potential differences between behaviours of fish treated with control seawater (current-day CO_2_ levels) and those treated with elevated levels of CO_2_ (mid and high CO_2_ treatments). Trials were carried out in 35 L aquaria (390×300×290 mm) and behaviour was recorded by video cameras to prevent observer-induced behavioural responses. A 50×50 mm grid was laid at the bottom of the aquarium and was submerged by a 10 cm hollow PVC pipe shelter placed at the centre of one end. This shelter had two exit holes which were facing the tank walls, with the curved edge of the pipe against the tank wall furthest from the video camera, which was placed on a tripod above the aquarium. Fish were placed in aquaria with aeration for an hour acclimation period prior to recording their behaviour. Trials lasted 20 minutes, during which time aeration was terminated and fish were not disturbed. Following the 20 minutes activity trial, 25 individual INVE Aquaculture Nutrition pellets were introduced to the upper left corner of the tank via a long PVC pipe, to ensure food was introduced in a novel manner and reactions were not a result of human conditioning. Feeding activity and behaviour were recorded for a further five minutes. Ten, eight and seven fish were videoed from the controls, mid and high treatments respectively. All trials were conducted in control seawater, as a pilot study and previous investigations [14]–[15] indicated that testing in control versus CO_2_ treated water has little to no effect on the results and that the effects of elevated CO_2_ last for a number of days when fish are placed in control water.

The first minute of each recording was excluded from analysis to control for any disturbance caused by the observer exiting the laboratory. The number of line crosses both away from the shelter (ie. parallel to the shelter) and across the tank (ie. perpendicular to the shelter were quantified from the videos. Two estimates of activity were calculated. First, total cumulative distance from shelter was calculated by tallying all the linear excursions during which the fish moved away from the shelter (i.e. tallying just the line crosses parallel to the shelter). Cumulative distance moved from shelter was used as an indication of boldness for these highly cryptic species. Second, the total number of line crosses (both parallel and perpendicular) were tallied as an indication of overall movement throughout the tank, not just away from the shelter. For the five minutes following the addition of food the time to respond to the presence of food and the activity levels (line crosses) were recorded. Time to respond to food was the time from the introduction of the food until the first feeding strike. Feeding strikes were identified as directed rapid movements toward the introduced food, whether they were successful bites or not.

### Data analysis

The proportion of time spent in either side of the two channel chamber was determined from the total time that fish were not located in the acclimation area at the back of the chamber. Dye trials indicated that some mixing of water sources occurred in the acclimation area. Therefore, any time fish spent in this small area was not indicative of a choice, and was excluded from analysis. To determine if predators exhibited a preference for prey skin extract, the mean proportion of time spent in the stream of water containing prey skin extract was compared against the null-expectation of 0.5 for no preference. The mean time that individuals spent in the chamber channel with the prey skin extract was then compared among the control seawater and the two CO_2_ treatments, using a one-factor analysis of variance (ANOVA) and a post-hoc Tukey's HSD means comparison tests. Assumptions of normality and homogeneity of variance were explored using residual analysis and deemed to be satisfied.

Movement and feeding behaviour were first compared among the three treatments with a multivariate ANOVA (MANOVA). One-factor ANOVAs with planned comparisons were then used to further explore any significant differences in behaviours between the CO_2_ treated fish and non-treated fish. Residual analysis was used to determine whether data were normally distributed and homogenous in variance. A square-root transformation was applied in cases where data did not meet the assumptions of MANOVA.

## Results

### Response of Pseudochromis fuscus to prey skin extracts

**Figure 1:**
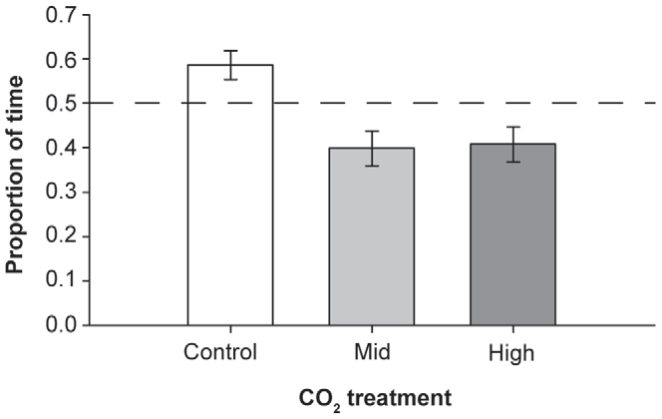
Response of *Pseudochromis fuscus* to prey skin extracts. Proportion of time (mean ± SE) spent in the channel receiving the prey skin extracts for *P. fuscus* exposed to control (n = 10), mid CO_2_ (n = 10) and high CO_2_ (n = 14) water treatments. Letters above bars indicate post-hoc Tukey's HSD groupings of means at p<0.05.

Predators spent approximately equal proportions of time in both channels of the chamber when no additional odour was present (average proportions; Control = 0.47±0.08, Mid = 0.47±0.10, High = 0.50±0.10); Fig. 1). *P. fuscus* treated in control seawater exhibited an attraction to the prey skin extract, spending significantly more than half their time in the compartment containing this cue (t = 2.621, df = 9, p = 0.028). In contrast, *P. fuscus* treated in CO_2_ enriched water spent significantly less than half their time in the channels containing prey skin extract (Mid = 2.577, df = 9, p = 0.030; High = 2.296, df = 13, p = 0.039). *P. fuscus* exposed to CO_2_ enriched water spent approximately 20% less time in the water stream containing prey odor compared with control fish (F_2,31_ = 6.854, p = 0.003; Fig. 1).

### Effects on activity levels and feeding behaviour of *Pseudochromis fuscus*

**Figure 2:**
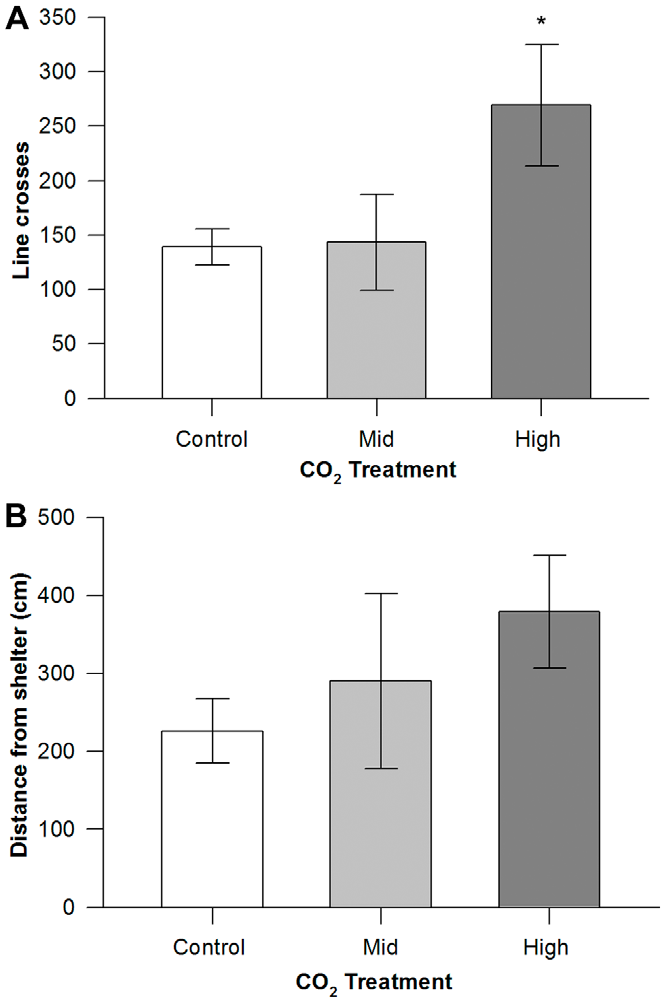
Movement behaviour of *Pseudochromis fuscus*. A. Mean (± SE) activity levels (denoted by line crosses) and B. mean cumulative distance moved from shelter for *P. fuscus* following exposure to control (n = 10), mid CO_2_ (n = 8) and high CO_2_ (n = 7) water treatments. Significance from planned comparisons (control vs. high) at p<0.05 = *.

Treatment in CO_2_ enriched water had a significant effect on movement patterns of *P. fuscus* (Pillai's trace_4,44_ = 0.412, p = 0.035; Fig. 2). *P. fuscus* in the high CO_2_ treatment displayed activity levels double that of fish kept in control seawater, crossing 269.14±55.76 (mean ± SE) lines compared to 139±16.54 for the control fish (F_2,22_ = 3.459, p = 0.049 Fig. 2a). *P. fuscus* in the mid CO_2_ treatment showed activity levels (143.50±43.97 lines) similar to that of the control fishes. The cumulative distance moved away from shelter by *P. fuscus* in all treatments was not significantly affected by CO_2_ treatment (F_2,22_ = 0.979, p = 0.39). However there was a trend for *P. fuscus* exposed to the elevated CO_2_ treatments to move a greater cumulative distance than *P. fuscus* in kept in control seawater (Fig. 2b).

**Figure 3:**
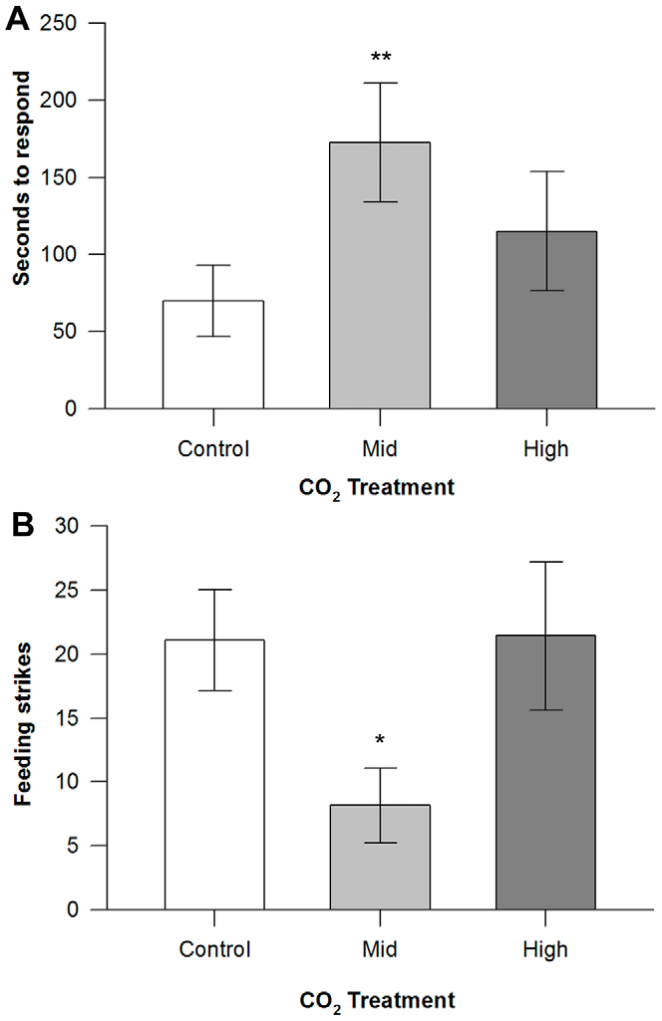
Feeding behaviour of *Pseudochromis fuscus*. A. Time to respond (seconds; mean ± SE) and B. feeding strikes (mean ± SE) of *P. fuscus* following exposure to control (n = 9), mid CO_2_ (n = 7) and high CO_2_ (n = 7) water treatments. Significance from planned comparisons (control vs. mid CO_2_) at p<0.01 = *, p<0.001 = **.

The feeding behaviour of *P. fuscus*, was significantly affected by treatment in CO_2_ enriched water (Pillai's trace_4,44_ = 0.402, p = 0.047; Fig. 3). Time taken to respond to the introduction of food to the tank was significantly greater for the mid CO_2_ treated fish (172.43±38.52 sec) when compared to the controls (41.10±14.70 sec; F_2,21_ = 4.616, p = 0.022; Fig. 3a), resulting in a delay in reaction by an average of two minutes. The effect of the high CO_2_ treatment on time to respond to food was less severe, with reaction times of just over one minute (65.9 seconds) slower than control fish. The feeding strikes followed similar patterns; *P. fuscus* in the mid CO_2_ treatment exhibited significantly fewer (8.14±2.91) feeding strikes compared to control (21.1±3.96) and high CO_2_ treated fish (21.43±5.80; F_2,21_ = 3.504, p = 0.05; Fig. 3b).

## Discussion

In this study, the first to investigate the effects of ocean acidification on the behaviour of a predatory fish, we show that the ability of a common meso-predator to detect chemical cues produced by it's prey is hindered and activity levels are elevated by exposure to elevated CO_2_. *P. fuscus* naturally exhibited a preference for the olfactory cues of injured prey, however, following exposure to dissolved CO_2_ concentrations that could be widespread in the ocean by the end of the century [7], they displayed a slight avoidance to these cues . Predators treated in control water spent approximately 60% of their time in the chamber channel containing prey skin extracts, but this value fell to around 40% in CO_2_ treated fish. A change in reaction to olfactory cues is consistent with previous studies that have found that larval fish exposed to elevated CO_2_ were unable to discriminate between ecologically important cues, becoming attracted to cues they normally avoided [15], [18] and exhibiting reduced preference for favourable cues [14]. This diminished preference could indicate a reduced ability to recognise the olfactory cues presented and is supported by the observation that CO_2_ treated *P. fuscus* were more inquisitive and explored the entire chamber, compared to the more directional response towards the cue exhibited by control fish.

*P. fuscus* inhabit topographically complex reef habitats, potentially limiting the effectiveness of locating prey via visual information over long distances [42]. Olfactory cues are suggested to be important in initiating a foraging response in predators, with the release of the prey skin extract resulting in a directional movement of predators towards prey [37]–[38]. A shift from attraction to repulsion of favourable prey cues due to ocean acidification could result in a decrease in the predatory activity of meso-predators and a reduced ability to respond to fluctuations in food availability.

The potential exists for effects of elevated CO_2_ on predator behaviour to counteract the increased risk of mortality posed to prey species. It is possible that reduced attraction of predators to damage-released skin extracts of prey, or even slight avoidance of the cue, could enhance the survival of prey. However, previous research addressing the effects of elevated CO_2_ on newly-settled prey fishes indicates a dramatic switch from complete avoidance to strong preference for predator odour following exposure to CO_2_ enriched water [15]. In contrast, the present study found that following CO_2_ treatment predators spent 20% less time in a water stream containing the smell of injured prey.

Although both predator and prey are affected by the elevated CO_2_, the repercussions of the loss of predator avoidance by the settling fish is likely to be more detrimental for prey survival than can be offset by a small reduction in predator response to prey olfactory cues. This supports earlier suggestions that ocean acidification is a potential threat to replenishment of reef fish populations [14]. Further investigations addressing the loss of olfactory information coupled with other traits that are important in the outcome of predator-prey interactions, such as other sensory modalities, boldness and locomotor performance, would provide a more detailed indication of the likely impacts to future reef fish populations.

Avoidance of the beneficial olfactory cue of injured prey may be attributed to an alteration of neuro-sensory functioning following exposure to elevated CO_2_ [14], [17]–[18].

The inability to distinguish between important olfactory cues, such as predator and non-predator odour [15], and reduced attraction to prey skin extract shown in this study, could be caused by impaired transfer of chemosensory signals across the olfactory epithelium or some other disruption to the olfactory nervous system. Changes in activity levels and feeding behaviour, which are not necessarily associated with olfactory sensitivity, suggest that the physiological mechanisms responsible occur at a cellular level within the nervous system. For example, cellular changes caused by acid-base regulation in CO_2_ exposed fish might affect neuronal pathways that mediate a range of functions, including olfactory discrimination and activity levels [17]. Detailed physiological studies would be required to test the possible mechanisms responsible for changes in olfactory responses and behaviour observed here and in previous studies.

A similar reduction in response to alarm cues has been described in freshwater fishes exposed to weakly acidic conditions [43]–[45]. However, the mechanisms involved are different to those responsible for the changes in behaviour observed here for *P. fuscus* and in larval fish exposed to elevated CO_2_. In the freshwater examples, exposure of the alarm cue to mineral acid (e.g. H_2_SO_4_) causes a non-reversible structural change in the alarm cue molecule, rendering it unrecognizable to the olfactory system [43]. In contrast, we presented the alarm cue in control water and it elicited a response in control fish as expected. Consequently, the changed behaviour of CO_2_ treated *P. fuscus* was due to their exposure to CO_2_, not a change in the alarm cue itself. Furthermore, trials associated with another study [46] demonstrated that presenting the same prey skin extracts used here in either control or CO_2_-enriched water elicited the same strong avoidance behavior in larval damselfishes (DM Dixson, unpublished data] indicating that elevated CO_2_ does not cause a chemical change in the cue in saltwater. However, it should be noted that this particular experiment does not address the possibility that in acidified water the prey alarm cues utilized could be subject to structural changes. Hearing is also affected by elevated CO_2_, indicating responses to CO_2_ are not confined to the olfactory system [17]. This is further evidence that the various behavioural effects observed are not simply due to a change in the structure of chemical cues. Nevertheless, it is intriguing that two different acidification processes both affect behavioral responses of fish to alarm cues, with potentially important consequences in freshwater and marine ecosystems.

Enhanced activity levels and altered feeding responses of *P. fuscus* are similar to the findings of the effects of elevated CO_2_ on newly settled fish. Munday and colleagues [14] found that following exposure to elevated CO_2_ *Pomacentrus wardi* were bolder, move further from shelter, were more active and less responsive to threats when compared to control individuals. Given that *Pseudochromis fuscus* themselves may be prey to larger predators, incidental movement further from shelter due to altered behaviour could increase their vulnerability. This effect however, remains to be assessed in the field to determine if changes to activity levels influence exposure of *P. fuscus* to larger predators.

The olfactory abilities and activity levels of fishes have previously been investigated, however this is the first demonstration of the direct influence of elevated CO_2_ on a predator's response to food. Delayed feeding response of *P. fuscus* in mid CO_2_ could be attributed to their inability to detect the presence of food due to the disruption of their olfactory capabilities. For *P. fuscus* in the high CO_2_ treatment, a faster response than the mid CO_2_ treated fish and a similar number of feeding strikes to the control fishes might have occurred as a result of higher activity levels and greater behavioural boldness, which would facilitate food detection. Predatory fish use a variety of sensory cues to detect and locate prey, and although olfaction is often important [31], vision can also play an important role in prey detection and capture [47]. Increased activity in the high CO_2_ treatment could have resulted in the fish relying more on vision than olfaction to detect food. Consequently, a consideration of all sensory abilities and behaviour, not just olfaction, will be important in determining the outcome of predator-prey interactions under acidified conditions. In one of the only other studies to investigate the potential effects of ocean acidification on predator-prey interactions, Bibby and colleagues [16] found that reduced shell thickness in the intertidal gastropod *Littorina littorea* at low seawater pH was associated with increased predator evasion behavior, which might help offset the increased risk of mortality in thin-shelled individuals. Therefore, the outcome of predator-prey interactions under changed environmental conditions can be complex and difficult to predict.

This study was carried out in laboratory conditions and thus, does not allow for a full evaluation of the potential effects of ocean acidification on the predatory ability and vulnerability of *P. fuscus* in their natural environment. Nevertheless, the effects observed suggest that the outcome of predator-prey interactions could be influenced by elevated *p*CO_2_ in nature. Furthermore, as these were short-term experiments involving acute exposure to elevated CO_2_, the potential for fish to acclimate or adapt to rapid ocean acidification was not tested. Munday and others [14] found that the settlement-stage fish exposed to elevated CO_2_ for four days exhibited almost identical impairment of their olfactory-mediated behavior as fish reared from birth at elevated CO_2_. Consequently, it does not seem likely that further increasing the duration of the CO_2_ treatments by days to weeks would have affected the results of this experiment. Long-term rearing experiments over months to years will be required to determine if either predators or prey can acclimate to elevated CO_2_ in the longer term. Munday and colleagues [14] also found that larval fish exhibited considerable variation in responses to acidification at approximately 700 ppm CO_2_, which suggests some capacity for selection of tolerant phenotypes. In contrast, *P. fuscus* did not exhibit an increase in individual variation in sensitivity at intermediate CO_2_ levels, even though the CO_2_ concentration (∼650 µatm) was similar to that at which variation in responses was observed in larval fishes.

This study is the first to demonstrate the potential impacts of ocean acidification on a predatory fish, beyond the early life history stages. Combined effects of elevated CO_2_ on attraction to prey odour and changes in general activity levels suggest that even moderate increases in atmospheric CO_2_ affect the behaviour of meso-predators and the outcome of interactions between *P. fuscus* and their prey. Further investigations are required to determine and test the effects of changed behaviour on predator success in the field and the impact this has on prey populations. However, the extreme attraction to predator odour by larval fishes under acidified conditions, compared to the relatively small avoidance of prey cues by meso-predators detected in this study suggest that it is unlikely that negative effects on predators will fully compensate for the increase in mortality rates of larval fish returning to the reef in an acidified ocean.
